# Rate and risk factors of metabolic components and component combinations according to hypertension status in Tibetans in a cross-sectional study

**DOI:** 10.1097/MD.0000000000031320

**Published:** 2022-10-28

**Authors:** Jihong Hu, Brian Thompson, Shuxia Wang, Minhao Guo, Chunjuan Yan, Fengfeng Ding, Peng Guo, Li Chen, Zhuoma Cao, Jianzong Wang

**Affiliations:** a Public Health School, Gansu University of Chinese Medicine, Lanzhou, China; b Department of Environmental Health Sciences, Yale University School of Public Health, New Haven, CT; c Affiliated Hospital of Gansu University of Chinese Medicine, Gansu, China; d Xiahe People’s Hospital, Gannan, China; e Tibetan Traditional Medical School, Gansu University of Chinese Medicine, Gannan, China.

**Keywords:** hypertension, metabolic component and combination, metabolic syndrome, normotension, Tibetan

## Abstract

To estimate the prevalence of metabolic syndrome (MS) and metabolic components and their associated factors and component combinations according to hypertension status in Tibetans living at high altitude. Multistage sampling of 1473 participants (799 hypertensive patients and 674 normotensive subjects). MS prevalence and the number of metabolic components ≥ 3 were significantly higher in the hypertensive than normotensives. In hypertensive patients, the most common component was central obesity and it combined with: high blood pressure, in those with 2 risk factors, plus fasting hyperglycemia, in those with 3 risk factors, and high triglyceride, in those with 4 risk factors. In normotensive subjects, the most common single component was low high-density-lipoprotein cholesterol, and most component combination included central obesity and hyperglycemia in those with 2 risk factors, plus high blood pressure in those with 3 risk factors, and high triglycerides in those with 4 risk factors. Body mass index and female both were associated with increased possibilities of MS in hypertensive and normotensive participants. Low incoming, and high educational levels were associated with an elevated probability of MS in normotensive Tibetans also. The priority of prevention from cardiovascular diseases by targeting metabolic components in the hypertensive was different from normotensives. Different MS components had various lifestyle and socioeconomic factors.

## 1. Introduction

Metabolic syndrome (MS) is characterized by a cluster of cardiometabolic abnormalities that include abdominal obesity, hyperglycemia, elevated blood pressure (BP), and dyslipidemia. MS has become a major public health challenge in both developed and developing countries.^[1]^ MS risk factors act synergistically to increase the risk of adverse cardiovascular events, including coronary artery disease and congestive heart failure and are associated with high cardiovascular morbidity and mortality.^[2]^ They have even been shown to increase the risk of all-cause mortality in the general population.^[3–7]^ Further, the larger the number of the MS components, the greater the risk of having cardiovascular diseases (CVDs).^[8]^ The presence of a greater number of MS risk factors may be more important than the diagnosis of MS in predicting subclinical atherosclerosis,^[9]^ the risk of CVDs,^[10]^ and CVDs mortality.^[11]^ However, not all of the individual components of MS were shown to contribute to the increased risk of all-cause mortality.^[6]^ Furthermore, the risk of cardiovascular disease is related to different combinations of MS components.^[12]^ These patterns are observed in both high- and low- income populations living at low altitude. However, studies from high altitude populations are scarce. This is concerning considering that world-wide, more than 17 million people live above 3500 m.^[13]^

Tibetans are known as one of the oldest high altitude native populations in the world. In China, Tibetans have the highest incidence of stroke.^[14]^ Prominent metabolic features, such as elevated BP, are higher among Tibetans than the rest of the Chinese population.^[15]^ Furthermore, these metabolic features have also increased substantially for all age groups over the past few decades.^[15,16]^ Most studies of CVD risk factors have been performed in urban Han populations. A small number of limited epidemiological studies have been done in Tibetan populations.^[17–22]^ These studies have been limited by either using the China Diabetes Society (CDS) diagnostic criteria,^[18–20,22]^ looking at only stage 1 hypertensive patients,^[19]^ or only investigating the relationship between salt sensitivity and MS^[20]^ or small sample in Tibetan Buddhism immigrants.^[22]^ Only one low-response study reported the risk of MS factors and the overall prevalence of MS by the IDF (International Diabetes Federation) diagnostic criteria.^[17]^ Risk of MS factors and the overall prevalence of MS were found to be lower, but some individual metabolic components (fasting hyperglycemia, abdominal obesity, and high BP) were higher, in Tibetan than other native populations.^[17]^ However, it is unclear if, among Tibetans, there is MS component heterogeneity and the degree to which this heterogeneity exists. This study was undertaken to estimate the prevalence of MS, MS metabolic components and common individual metabolic component combinations, and their associated factors in hypertensive and normotensive Tibetans.

## 2. Materials and Methods

### 2.1. Study population

A case-control study on salt sensitivity and hypertension was conducted among adult Tibetans in the Tibet Autonomous Region of Gannan from August 2013 to September 2014.^[20]^ The study was approved by the Gansu University of Chinese Medicine ethics committee (Lanzhou, China) (2013-02) and written informed consent was obtained from participants before the investigation. The sample size was calculated through an online-software, OpenEpi with a 95% confidence interval (CI), 80% power, underestimated exposure rate of case (33.8%) and overestimated exposure rate of control (26%).^[23,24]^ A total of 1144 Tibetans, including 572 cases and 572 controls were required after taking into account a 10% non-response rate. This case-control study was based on a sampling survey. The details of sampling were as following. Firstly, we sampled in two randomly selected counties, Xiahe and Hezuo, where the altitude is between 3500 and 4000 m. All the native Tibetan residents aged ≥ 18 years, with at least 3 generations of paternal ancestry within this ethnic group were recruited from the lists of residents which were provided by village governments, using a stratified, multistage sampling method. After selecting Xiahe and Hezuo counties in the initial stage, five villages from each of the two counties were randomly selected during the second stage. Finally, people without self-reported secondary hypertension were invited to participate in the survey. Participants were classified as either hypertensive or normotensive subjects according to self-reported hypertension history or an average of systolic BP ≥ 140 mm Hg and/or diastolic BP ≥ 90 mm Hg. A total of 1473 Tibetans who were randomly sampled from this sampling survey took part in this investigation, including 799 hypertensive patients, in the case group, and 674 normotensive subjects, in the control group. Data for this study was based on the aforementioned case-control study.

### 2.2. Data collection

We conducted the majority of the survey at the village committee’s office and made household visits for participants who lived far from the office. Demographic characteristics, past medical history, and lifestyle-related factors were collected through face-to-face interviews using a standard questionnaire administered by interviewers trained in the Tibetan language. Collected information included demographical information (including gender, age, occupation (farmer and herdsman, workers, others), educational levels (no education, primary or less, and secondary or more), yearly family income (<3000 yuan/yr, 3000–4999 yuan/yr, or ≥5000 yuan/yr), and past medical history (including hypertension, diabetes mellitus (DM), and dyslipidemia), life-style risk factors (including exercise (no exercise, <3 times/wk, or ≥3 times/wk), current smoking (“yes” is defined by smoked ≥100 cigarettes in their lifetime and smoked in the last 28 days), and current drinking (answers range from three times a day or more often to less than once a month but at least once a year).

### 2.3. Physical examination and laboratory test

BP, weight, height, and waist circumference (WC) were measured with validated instruments according to standard operating procedures. Additionally, all participants took fasting glucose, total cholesterol, triglycerides (TG), high-density-lipoprotein cholesterol (HDL), low-density-lipoprotein cholesterol.

BP was measured from the right arm of each participant, with an appropriately sized cuff, in the seated position in a quiet room at normal room temperature. After a short rest period, a trained practitioner averaged BP across two readings. A validated electronic sphygmomanometer (Microlife 3BTO-A) was used to take the BP measurement. Body mass index (BMI) was calculated as the weight in kilograms divided by the height in meters squared. WC was measured at the level of the umbilicus while the participants were standing.

Serum lipids (total cholesterol, TG, HDL-C, and low-density-lipoprotein cholesterol) and plasma fasting glucose were tested with the HITACHI 7180 Chemistry Analyzer (HITACHI Company, Japan) in a certificated laboratory.

### 2.4. MS definition

According to the 2005 IDF definition, for a person to be defined as having MS they must have:

Central obesity (defined as WC ≥ 90 cm for Asian men and ≥80 cm for Asian women)

Plus any two of the following four factors:

(1) Raised TG level: ≥150 mg/dL (1.7 mmol/L), or specific treatment for this lipid abnormality
(2) Reduced HDL: <40 mg/dL (1.03 mmol/L) in males and <50 mg/dL (1.29 mmol/L) in females, or specific treatment for this lipid abnormality
(3) Elevated BP: systolic BP (SBP) ≥ 140 or diastolic BP (DBP) ≥ 90 mm Hg, or treatment of previously diagnosed hypertension
(4) Raised fasting plasma glucose (FPG) ≥ 100 mg/dL (5.6 mmol/L) or previously diagnosed type 2 diabetes.

### 2.5. Statistical methods

All analyses were performed with the SPSS 13.0 statistical package (SPSS Inc., Chicago, IL). The chi-square test or Mann–Whitney *U* test was used to examine the differences of quality variables between hypertensive and normotensive subjects. To examine the differences of quantity variables between hypertensive and normotensive and MS and non-MS subjects, either a Student’s *t* test, for normally distributed data, or a Mann–Whitney *U* test, for non-normally distributed data, were used. Logistic regression analysis was used to examine the independent contributions of age, gender, occupation, educational level, smoking, drinking, income, and physical activity/exercise to MS and metabolic components and common metabolic components combinations. We also checked for interactions between age, gender, income and education with the outcome. However, we did not find any evidence of an interaction.

## 3. Results

**Figure 1.**
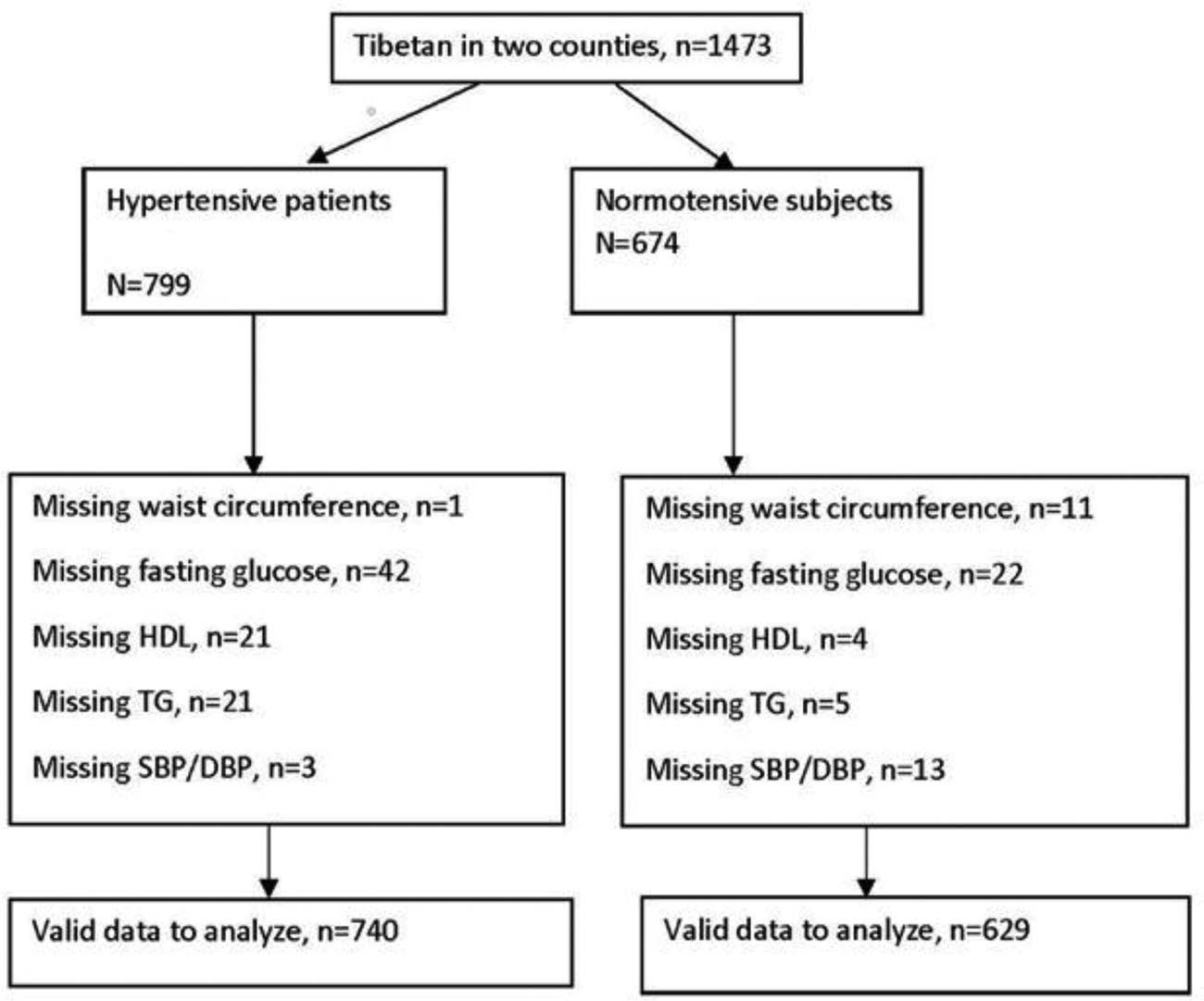
Flow chart of the participants in the original case control study. DBP = diastolic blood pressure, HDL = high-density-lipoprotein, SBP = systolic blood pressure, TG = triglycerides.

1369 (92.9%) out of 1473 participants completed the laboratory data needed to identify the presence of MS were valid for analysis (Fig. 1). 92.6% of hypertensive patients and 93.3% of normotensive subjects had complete data for analysis.

### 3.1. The characteristics of subjects

There were no significant differences between participants with complete data and those with missing value (Table 1). In comparison with normotensive subjects, hypertensive patients were significantly older, less likely to be female or exercise, and have lower current smoking and drinking rates. However, they did have higher BMI, WC, FPG, TG, HDL, SBP, and DBP. In hypertensive patients, subjects with MS had significantly higher BMI, WC, FGP, TG, HDL, and SBP and but had a lower income, when compared to subjects without MS. In normotensive subjects, individuals with MS were significantly more likely to be female and had elevated rates of FPG, TG, and HDL, but had lower income, current smoking, and drinking rates, when compared to normotensive subjects without MS.

**Table 1:** The characteristics of subjects with and without hypertension.

	Hypertensive patients (n = 799)				Normotensive patients (n = 674)			
	Valid (n = 740)			Missing (n = 59)	Valid (n = 629)			Missing (n = 45)
	MS+ (n = 406)	MS− (n = 334)	Total		MS+ (n = 100)	MS− (n = 529)	Total	
Age (yr)	57.1 ± 10.9*	58.3 ± 11.0	57.6 ± 11.0*	56.1 ± 12.1*	46.6 ± 9.1	44.6 ± 9.1*	44.8 ± 9.1	46.5 ± 8.7
Sex (female, %)	51.7*	46.7	49.5*	48.6*	79.0	53.1†	56.9	55.3
Educational level								
No education	76.3*	73.6	69.3*	57.0	60.0	63.7*	62.8	54.8
Primary school or less	12.5	12.2	17.5	24.4	25.0	23.0	23.5	22.6
Secondary school or more	11.3	14.3	13.2	18.6	15.0	13.3	13.7	22.6
Income (yuan/year)								
<3000	73.8*†	69.4	72.9*	67.8	83.8	72.1*†	74.1	74.2
3000–4999	12.6	10.3	17.9	20.5	16.2	27.0	25.2	22.6
≥5000	13.6	20.3	9.3	11.7	0	1.0	0.8	3.2
Occupation								
Farmer and herdsman	87.3	86.2	86.5	87.1	85.0	85.6	85.6	77.4
Other jobs	12.7	14.0	13.8	12.9	15.0	14.2	14.4	22.6
Exercise								
No exercise	11.6*	9.3	26.0*	34.2*	39.5	46.2*	45.1	46.2
≤3 times/wk	42.3	38.3	31.0	31.6	15.1	20.3	19.4	12.4
>3 times/wk	46.0	52.5	43.0	34.2	45.4	33.5	35.4	41.4
Current smoking	13.4	10.5	12.2*	13.6*	14.4	36.8*†	33.4	35.5
Current drinking	10.9	16.9	15.9*	16.9*	10.3	28.6*†	25.4	26.9
BMI (kg/m^2^)	27.9 ± 3.6*†	25.4 ± 3.4	26.8 ± 3.7*	26.3 ± 3.4*	24.1 ± 2.6	23.5 ± 2.9*	23.6 ± 2.8	24.7 ± 3.6
WC (cm)	99.8 ± 11.6*†	90.5 ± 11.7	95.6 ± 12.5*	93.6 ± 14.4*	90.4 ± 9.2	88.3 ± 13.8*	88.6 ± 13.2	86.0 ± 12.2
FPG (mmol/L)	5.4 ± 1.4*†	4.8 ± 0.9	5.1 ± 1.3*	5.1 ± 1.4*	6.3 ± 1.6	5.8 ± 1.4*†	5.8 ± 1.5	5.4 ± 0.8
TG (mmol/L)	2.7 ± 1.5*†	1.6 ± 0.9	2.2 ± 1.4*	2.2 ± 1.2*	2.1 ± 1.2	1.2 ± 0.9*†	1.3 ± 1.0	1.4 ± 1.1
HDL (mmol/L)	1.6 ± 0.5*†	1.7 ± 0.4	1.6 ± 0.5*	1.5 ± 0.4*	1.4 ± 0.6	1.8 ± 0.8†	1.7 ± 0.8	1.6 ± 0.5
SBP (mmol/L)	160.9 ± 17.4*†	157.9 ± 16.6	159.6 ± 17.1*	158.2 ± 22.7*	127.2 ± 14.3	125.8 ± 14.1*	126.1 ± 14.2	125.6 ± 8.8
DBP (mmol/L)	101.2 ± 9.5*	99.9 ± 8.5	100.6 ± 9.1*	102.4 ± 9.7*	80.5 ± 9.6*	79.7 ± 9.0*	79.8 ± 9.1	79.9 ± 6.6

BMI = body mass index, DBP = diastolic blood pressure, FPG = fasting plasma glucose, HDL = high-density-lipoprotein cholesterol, MS− = subjects without metabolic syndrome, MS+ = subjects with metabolic syndrome, SBP = systolic blood pressure, TG = triglycerides, WC = waist circumference.

*P < .05, hypertensive patients compared with normotensive subjects.

† *P* < .05, MS+ compared MS−.

### 3.2. The prevalence of MS and common metabolic components in hypertensive and normotensive participants

**Figure 2.**
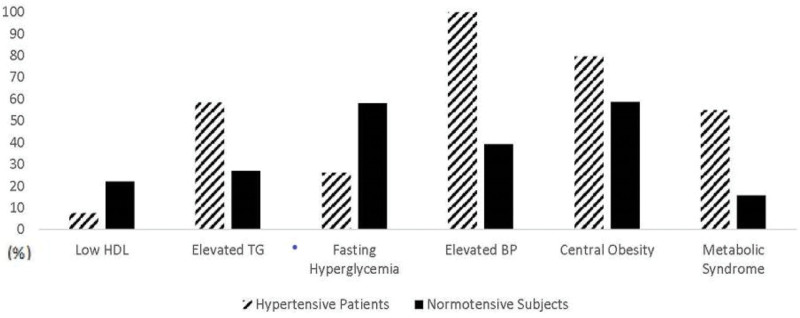
Prevalence of MS and its components between hypertensive patients and normotensive subjects. Significant differences in the prevalence of MS and its components exist among hypertensive patients and normotensive subjects (all *P* < .001). BP = blood pressure, HDL = high-density-lipoprotein, IDF = International Diabetes Federation, MS = metabolic syndrome, TG = triglycerides.

The overall prevalence of MS and most of its components were significantly higher in hypertensive patients than in normotensive subjects (Fig. 2). In hypertensive patients, central obesity (79.5%) and high TG (58.4%) were common components of MS. Low HDL and fasting hyperglycemia were found to be more frequent in normotensive subjects than in hypertensive patients (all *P* < .01).

In normotensive subjects, the female had higher MS and low-HDL, however, lower central obesity and high-TG than the male, and the prevalence of MS increased with age. In hypertensive patients, the female had higher low HDL and central obesity, lower high TG than the male (Table 2).

**Table 2:** Prevalence of MS and MS profiles in hypertensive patients and normotensive subjects across different age groups and gender groups (%).

	Low HDL	High TG	Central obesity	Fast hyperglycemia	High BP	MS
Hypertensive patients						
Gender						
Male	5.1*	64.1*	68.6*	28.9	–	48.3
Female	9.8	52.1	89.3	23.9	–	51.7
Age (yr)						
<40	13.3	61.2	73.9	18.6	–	51.2
40-	7.5	58.1	84.1	25.0	–	61.9
50-	7.3	58.6	78.6	25.1	–	56.3
≥60	6.5	57.3	77.2	28.4	–	51.9
Normotensive subjects						
Gender						
Male	7.5*	32.0*	64.4*	59.4	13.7	7.6*
Female	33.6	23.5	24.0	56.6	9.1	21.7
Age (yr)						
<40	20.8	22.2	59.0	52.8	14.7	12.3†
40-	24.9	31.0	56.0	60.6	21.3	15.20
50-	20.6	27.8	60.6	58.2	27.2	19.8
≥60	16.0	45.8	44.0	79.2	6.2	21.7

BP = blood pressure, HDL = high-density-lipoprotein cholesterol, MS = metabolic syndrome, TG = triglycerides.

*P < .05, the female is significantly different from the male.

† *P* < .05 significantly positively related with age.

### 3.3. The number of metabolic components and component combinations in hypertensive and normotensive participants

**Figure 3.**
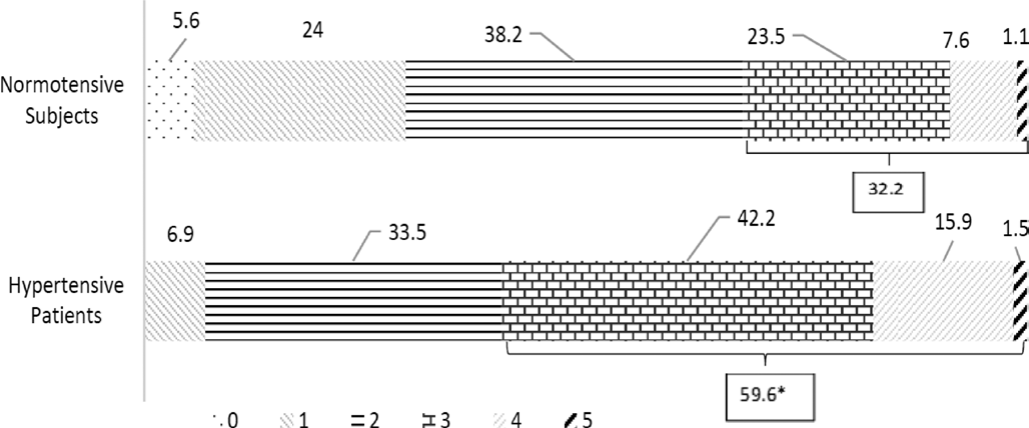
Number of metabolic disorders between hypertensive patients and normotensive subjects (%). *P < .05, hypertensive patients are significantly greater than normotensive subjects.

**Figure 4.**
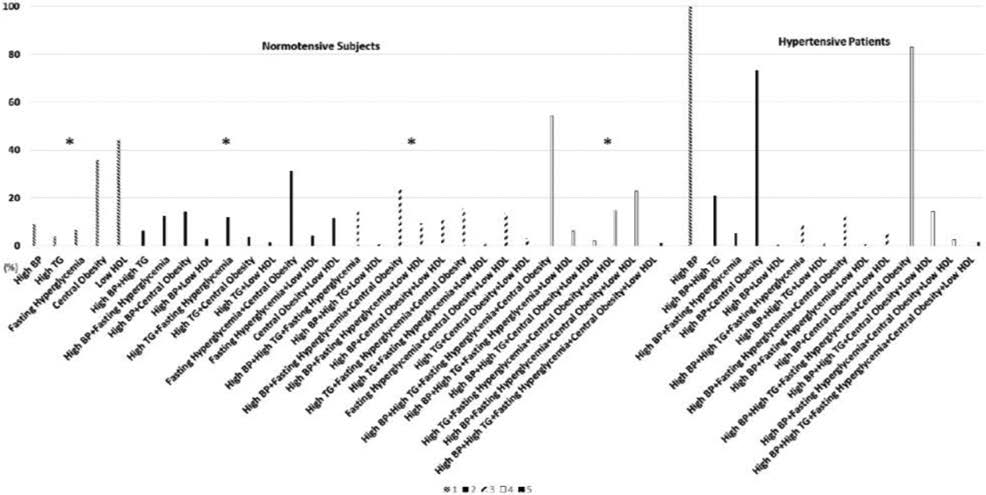
Metabolic components combinations according to the number of metabolic risk factors (%). *The individual components combinations were significantly different among normotensive subjects and hypertensive patients with the same number of metabolic disorders.

In hypertensive subjects, the prevalence rates of more than three components of MS (≥3) were significantly higher than in normotensive subjects (59.6% vs 32.2%, *P* < .001) (Fig. 3). Further, the individual component combinations in hypertensive patients were significantly different from normotensive subjects with the same number of metabolic disorders (all *P* < .05) (Fig. 4). The numbers of individual component combinations with more than 2 disorders was less in hypertensive patients than normotensive subjects. Among the subjects with only one metabolic component, the most common component was low HDL (44.4%) in normotensive subjects and high BP (100.0%) in hypertensive patients. Among the subjects with 2 metabolic components, the most common component combination was fasting hyperglycemia plus central obesity (31.3%) and hypertension plus central obesity (73.4%) in normotensive and hypertensive subjects, respectively. Among the subjects with 3 metabolic components, the most common component combination, for both hypertensive patients (12.5%) and normotensive subjects (23.6%), was high BP plus fasting hyperglycemia and central obesity. But the component combination with high BP plus high TG and central obesity was not founded both in hypertensive and normotensive subjects. Among the subjects with 4 metabolic components, the most common component combination was high BP plus high TG, fasting hyperglycemia, and central obesity for both normotensive and hypertensive subjects (54.2% vs 83.1%, *P* < .01). Nevertheless, the rate of 5metabolic components was similar in hypertensive patients and normotensive subjects (1.5% vs 1.1%, *P* > .05).

### 3.4. The odd ratios and 9595% CIs of associated factors of MS and its components

In normotensive subjects, age is positively associated with high BP and MS (*P* < .05); BMI is positively related to central obesity (*P* < .05); female subjects were more likely to have lower HDL and triglyceride levels by comparison to males (*P* < .05); having a high education level was associated with a reduced possibility of high BP (*P* < .05), but with an increased probability of MS (*P* < .05); having a moderate income level was related with a decreased probability of MS (*P* < .05); other jobs increased the risk of central obesity compared with farmer and herdsman (*P* < .05); more exercise was associated with an elevated possibility of high TG and central obesity (both *P* < .05) (Table 3). In hypertensive patients, sex (female) was related with an increased risk of central obesity (*P* < .05), but with a reduced probability of high TG (*P* < .05); BMI was positively associated with low-HDL, high-TG, fasting hyperglycemia, and central obesity (all *P* < .05); more exercise was associated with a decreased probability of low HDL (*P* < .05); current smoking was associated with an increased possibility of high TG (*P* < .05).

**Table 3:** ORs and 95% CIs according to metabolic components and metabolic syndrome among normotensive and hypertensive subjects.

	Low HDL	High TG	Fasting hyperglycemia	High BP	Central obesity	MS
Normotensive subjects						
Age (yr)	1.01 (0.98–1.04)	1.01 (0.99–1.03)	1.03 (1.01–1.05)	1.05 (1.03–1.07)*	1.01 (0.99–1.04)	1.04 (1.01–1.07)*
Gender						
Male	1.0	1.0	1.0	1.0	1.0	1.0
Female	5.31 (2.54–11.12)*	0.55 (0.31–0.96)*	0.89 (0.55–1.44)	0.71 (0.43–1.18)	10.22 (5.583–18.88)*	2.67 (1.26–2.63)*
BMI (kg/m^2^)	0.98 (0.90–1.07)	1.01 (0.94–1.08)	0.95 (0.89–1.01)	1.01 (0.95–1.08)	1.28 (1.18–1.40)*	1.11 (1.01–1.21)*
Education						
No education	1.0	1.0	1.0	1.0	1.0	1.0
Primary school or less	1.06 (0.58–1.94)	1.72 (1.04–2.84)*	1.24 (0.78–1.97)	0.80 (0.50–1.29)	1.52 (0.87–2.65)	1.91 (0.99–3.68)
Secondary school or more	1.58 (0.71–3.53)	1.03 (0.50–2.12)	1.41 (0.77–2.57)	0.48 (0.25–0.94)*	1.32 (0.63–2.78)	2.78 (1.18–6.54)*
Income						
Income < 3000 yuan/yr	1.0	1.0	1.0	1.0	1.0	1.0
Income 3000–5000 yuan/yr	0.89 (0.49–1.60)	0.93 (0.56–1.55)	0.71 (0.46–1.10)	1.09 (0.69–1.74)	0.73 (0.42–1.26)	0.47 (0.24–0.94)*
Income ≥5000 yuan/yr	*0*	0.79 (0.08–7.72)	0.48 (0.08–3.06)	1.21 (0.18–8.35)	0.88 (0.10–7.65)	0
Occupation						
Farmer and herdsman	1.0	1.0	1.0	1.0	1.0	1.0
Other jobs	1.11 (0.55–2.26)	0.58 (0.26–1.30)	1.07 (0.60–1.90)	1.33 (0.71–2.51)	4.39 (1.96–9.86)*	1.49 (0.67–3.32)
Exercise						
No exercise	1.0	1.0	1.0	1.0	1.0	1.0
≤3 times/wk	0.63 (0.34–1.18)	0.50 (0.25–1.01)	1.13 (0.69–1.84)	0.78 (0.46–1.32)	4.41 (2.26–8.62)*	0.86 (0.41–1.77)
>3 times/wk	0.61 (0.36–1.04)	2.60 (1.64–4.12)*	1.23 (0.981–1.87)	1.27 (0.83–1.95)	0.73 (0.44–1.20)	1.81 (1.02–3.23)*
Hypertensive individuals						
Gender						
Male	1.0	1.0	1.0	–	1.0	1.0
Female	2.01 (0.98–4.10)	0.66 (0.47–0.93)*	0.82 (0.55–1.20)	–	10.30 (5.79–18.31)*	1.45 (1.01–2.09)*
BMI	1.16 (1.07–1.26)*	1.09 (1.04–1.14)*	1.06 (1.01–1.11)*	–	1.54 (1.41–1.69)*	1.23 (1.17–1.30)*
Exercise						
No exercise	1.0	1.0	1.0	–	1.0	1.0
≤3 times/wk	0.35 (0.13–0.92)*	1.69 (0.96–2.97)	1.10 (0.59–2.04)	–	0.53 (0.22–1.26)	0.87 (0.51–1.48)
>3 times/wk	0.67 (0.27–1.64)	1.46 (0.84–2.55)	1.18 (0.64–2.19)	–	0.58 (0.24–1.38)	0.53 (0.26–1.06)
Current smoking						
N	1.0	1.0	1.0	–	1.0	1.0
Y	1.68 (0.57–4.97)	2.11 (1.13–3.92)*	1.05 (0.57–1.91)	–	0.80 (0.38–1.67)	1.40 (0.78–2.52)

Adjusted for age, gender, BMI, education, yearly income, occupation, exercise, current drinking and smoking in Logistic Regression analysis.

BMI = body mass index, BP = blood pressure, HDL = high-density-lipoprotein cholesterol, MS = metabolic syndrome, TG = triglycerides.

## 4. Discussion

Tibetans are one of the oldest high altitude natives in the world. In China, Tibetans have the highest rate of hypertension^[15]^ and have a rising trend for all age groups in the last decades.^[15,16]^ Hypertension is one of the most commonly identified components of the MS.^[25]^ When hypertension exists with other metabolic risk factors in an individual, they act synergistically to increase the risk of CVDs well above that which results from the sum of the individual risk factors.^[26]^ Recognition of this fact has led to a reorientation regarding risk stratification and management of hypertension. Accordingly, current guidelines on hypertension diagnosis and management emphasize that total CVDs risk should be quantified so that the type and intensity of treatment can be tailored to the degree of overall risk rather than the level of BP alone.^[27]^ In order to move towards a management approach, there must be both a search for and identification of multiple CVDs risk factors in patients.

To our knowledge, this study is the first to explore the MS and its components and the common metabolic component combinations separately in hypertensive patients and normotensive subjects among Tibetans from rural Gannan. The prevalence of MS by IDF in hypertensive (54.9%) and normotensive participants (15.6%) in this study were both higher than found by CDS in a recent study on Chinese adults (11.0%)^[28]^ and the overall prevalence of MS by CDS in Tibetans (26.9%) in our previous study^[18]^ also was higher than it in the overall Chinese by CDS,^[28]^ but lower than other ethnic groups by Adult Treatment panel III.^[29]^ This difference in prevalence may reflect both the disparities in components of MS between overall Chinese adults and Tibetans in the present study and different diagnostic definitions and different altitude. We found that the prevalence of MS by IDF was higher than it by CDS or Adult Treatment panel III in Stage 1 hypertensive Tibetans.^[19]^ Tibetans have a slightly greater mean BMI and TG levels than overall Chinese adults,^[28]^ however, mean HDL-C levels are slightly lower.^[28,29]^ Moreover, the unique lifestyle characterized by special local diets and hypoxia at high altitudes^[30,31]^ may partially account for the difference in prevalence. Nevertheless, the prevalence in this study is higher than the 8.2% observed in a 2010 study conducted on Tibetans in Lhasa^[17]^ and the 3.6% in a 2013 study conducted on Tibetan in Derong.^[22]^ The prevalence of MS among adults in China has recently shown an increasing trend.^[18]^ Tibetans are currently undergoing an epidemiological transition^[32]^ which may influence both health behaviors and outcomes at the population level.

It was further found that the prevalence of MS was higher among hypertensive patients (54.9%) than in normotensive subjects (15.6%), which is consistent with previous reports in China^[28,33,34]^ and other parts of the world.^[35,36]^ MS in hypertensive subjects was more prevalent than in the general population using the IDF criteria (43.1% vs 18.2%).^[33,34]^ It was reported that the prevalence of MS was twice as high in the hypertensive population compared to the normotensive population.^[36]^ Other studies have also found that MS was prevalent in hypertensive adults.^[37–40]^ A possible reason for our elevated prevalence of MS may be due to including older individuals with greater BMI, WC, TG, BP, and more males in our hypertensive participants than in our normotensive participants (Table 1 and Fig. 2). Age, sex, and elevated TG or TG/HDL ratio were shown to predominantly affect the MS.^[41]^ In Chinese people, it was reported that, individuals older than 40 years old, BMI is higher in women than men and increases with age before 70 years old.^[42]^ In this study, hypertensive participants were older than normotensive subjects (57.6 vs 44.8, *P* < .05). Furthermore, 74.6% of normotensive subjects were younger than 40 years old and 32.5% of hypertensive participants were younger than 40 years old. Moreover, hypertensive patients have been shown to have a higher frequency of central obesity, increased levels of TG, and elevated blood sugar.^[35]^ Higher prevalence of MS may imply that patients with hypertension tend to have more clustering of other metabolic abnormalities than normotensive individuals. In this study, the prevalence of more than one metabolic disorder was higher in hypertensive patients than in normotensive subjects (93% vs 70%, *P* < .05). All combinations exposed to individuals have different all-cause mortality risks.^[43]^ Therefore, it is necessary to explore specific MS component combinations according to the number of MS components.

To the best of our knowledge, this study is the first to demonstrate the metabolic components and their combinations in Tibetans. In the present study, the most common elements of MS component combinations were similar between hypertensive and normotensive participants (central obesity, fasting hyperglycemia, high BP and high TG) in the clustering of ≥3 disorders (seen in Fig. 4). Our results differed slightly from what other investigators have reported in Brazilians.^[17,35,44]^ The most combination with central obesity, hyperglycemia, high BP and low HDL was found in Brazilians.^[35,44]^ This disparity can be contributed to both dietary and ethnic differences. In China, it was reported that central obesity, high-BP, hyperglycemia, and low-HDL were the strongest risk factors of CVDs with ≥3 components combinations.^[12]^ However, in Tibetans, high-TG has a higher prevalence rate and a stronger correlation with coronary heart disease than low HDL.^[17]^ Additionally, we found that low HDL was the most frequent single component in normotensive subjects (Fig. 4) as it was reported in Chinese adults.^[45]^ However, the overall proportion of central obesity and fasting hyperglycemia were found to be higher than low HDL (Fig. 2). This suggests that central obesity and hyperglycemia easily combine with other metabolic disorders among normotensive subjects. Therefore, low HDL should be controlled when there is single metabolic disorder, while high TG should be controlled when dyslipidemia is combined with other metabolic disorders when hypertension didn’t present. Interestingly, in hypertensive subjects, the most common component was central obesity (79.5%) and it combined with: high BP, in those with ≥2 risk factors, plus fasting hyperglycemia, in those with ≥3 risk factors, and high TG, in those with 4 risk factors. This seems to suggest that central obesity is a core component of MS in hypertensive Tibetans. However, the component combination high BP plus high TG, and central obesity was not observed in either hypertensive or normotensive subjects with 3components combinations. This is despite the most common elements of MS component combinations being high BP, high TG, central obesity, and hyperglycemia in the clustering of ≥3 disorders in this study. This suggests that lacking hyperglycemia decreases the possibility of MS components combined with other disorders in Tibetans. Which metabolic component is critical in MS has been unclear, but the IDF belief is that central obesity is the most important. Moreover, in China, the prevalence of central obesity, DM, hypertension and dyslipidemia all rapidly increased from 2002 to 2012.^[46]^ Additionally, we found that hypertension increased the risk of MS and high TG, but decreased the risk of low HDL and hyperglycemia (Table 2). This result was consistent with the study in Bangladesh which showed that the mean HDL level was lower in the hypertensives compared to normotensives.^[45]^ Nevertheless, hypertension was positively associated with type 2 DM.^[48]^ It was reported that there was an inverse association between diabetes and altitude.^[49]^ Thus, the strategies for prevention, and early and effective approaches to minimize the possible negative impact from the association of MS and hypertensive disease should vary according to hypertension status in highlander Tibetans.

The study on the risk factors of metabolic components and MS was scarce in Tibetans. In this study, we found in normotensive and hypertensive participants, sex (female) and BMI both were associated with increased possibilities of central obesity and MS, but with decreased probability of hypertriglyceridemia (Table 3). In normotensive participants, sex (female) also was associated with an increased possibility of low HDL. It was reported that the prevalence of central obesity was significantly higher in women than men. In addition, females have more MS components than males^[34,45,50–52]^ including hypertensive adults from rural Northeast China^[34]^ and overall Chinese adults.^[55]^ In Tibetans, it was also reported that females have a significantly increased risk of MS and combination of central obesity, fasting hyperglycemia, high BP,^[17]^ and low HDL.^[53]^ In this study, BMI significantly positively affected both all of the components of MS and MS. These results are consistent with many previous studies.^[54–56]^ In this study, current smoking increased the risk of high TG among hypertensive patients. This result is discordant from a previous study in Tibetans.^[53]^ The use of different subjects (hypertensive Tibetans vs general Tibetans) may explain this difference. An unhealthy lifestyle and dyslipidemia may have a stronger negative influence on CVDs. Thus, hypertension accompanied with other metabolic components could be treated by improving modifiable lifestyle factors.

It is noted that normotensive participants had lower income and education level than hypertensive subjects (Table 1). Furthermore, a moderate income was associated with a decreased possibility of MS, but a high education level and special occupation (farming and herdsman) were related with increased probabilities of MS in normotensive subjects (Table 3). Most Tibetans in Gannan live in rural areas where subsistence farming and herding is the predominant occupation and there are both low education and income levels. However, they are currently undergoing socio-economic development and an epidemiological transition. Low education levels, in Sweden, have been associated with an increased risk of MS.^[57,58]^ Tibetans have a higher prevalence of hypertension, but lower rates of awareness, treatment, and control than the rest of China.^[15]^ With ongoing demographic changes and an aging population, the impact of the MS will be significantly greater in developing countries^[59]^ and Tibetans. Additionally, we found more exercise increased the risk of high TG and central obesity. Consciously changing behavior led to this result was different from other studies when patients knew their metabolic disorders and the risk of metabolic disorders.

This study has certain limitations. Firstly, this study was designed to explore the relationship between salt sensitivity and hypertension in Tibetans as a case-control study. Thus, the sample may not represent the population well. However, hypertensive and normotensive participants were both selected randomly from villages, with a stratified, multistage sampling method. Moreover, our sample size and response rate were larger than previous related studies in Tibetans.^[17,53]^ Furthermore, this paper aimed to explore the prevalence of MS respectively in hypertensives and normotensive population not prevalence of hypertension. Secondly, our regression models were adjusted for known risk factors derived from studies in low altitude populations. Thus, unmeasured confounding factors could bias our estimates. We not analyzed the dietary factors due to not collect information related. Additionally, despite some positive findings, the risk factors of MS can’t be confirmed in this study due to a cross-section study. A further prospective study will be needed. Lastly, some subjects were excluded from the data analysis due to missing data for some of the metabolic components. Our response rate was high at 92.6% in hypertensive patients and 93.3% in normotensive subjects. A non-significant difference in characteristics between subjects who were analyzed and participants enrolled with missing values indicates that our selection may not have substantially affected MS prevalence estimates.

## 5. Conclusion

The prevalence of MS, its components (hyperglycemia, obesity, high TG, and high BP) and having more than 3 MS components were higher in hypertensive Tibetans than normotensive Tibetans. In normotensive subjects, central obesity and hyperglycemia tended more likely to combine with other metabolic components than low HDL. In hypertensive patients, central obesity seemed to be critical. Different MS components had various lifestyle and socioeconomic factors in hypertensive and normotensive participants. The priority of prevention from CVDs by targeting metabolic components in the hypertensives was different from normotensives though the most frequent elements of MS components combinations (including central obesity, high BP, high TG, and hyperglycemia) were similar. Different MS components had various lifestyle and socioeconomic factors in hypertensive and normotensive participants in rural Tibet highlander. Our study underlined the value of questioning metabolic components and their individual combinations in hypertensive patients and normotensive subjects to identify individuals with high risk of CVDs.

## Author contributions

**Conceptualization:** Brian Thompson, Jianzong Wang.

**Data curation:** Shuxia Wang, Peng Guo.

**Formal analysis:** Shuxia Wang, Fengfeng Ding, Peng Guo, Li Chen.

**Funding acquisition:** Li Chen.

**Investigation:** Zhuoma Cao.

**Methodology:** Shuxia Wang, Chunjuan Yan, Fengfeng Ding.

**Project administration:**.

**Resources:** Minhao Guo.

**Writing – original draft:** Jihong Hu.

**Writing – review & editing:** Jihong Hu.
